# Screening for frailty phenotype with objectively-measured physical activity in a west Japanese suburban community: evidence from the Sasaguri Genkimon Study

**DOI:** 10.1186/s12877-015-0037-9

**Published:** 2015-04-02

**Authors:** Sanmei Chen, Takanori Honda, Tao Chen, Kenji Narazaki, Yuka Haeuchi, Atin Supartini, Shuzo Kumagai

**Affiliations:** Department of Health Behavior and Sciences, Graduate School of Human-Environment Studies, Kyushu University, Kasuga City, Japan; Faculty of Arts and Science, 6-1 Kasuga koen, Kasuga City, Fukuoka Prefecture 816-8580 Japan

**Keywords:** Frail older people, Aging, Prevalence, Accelerometer, Community health

## Abstract

**Background:**

The low physical activity domain of the frailty phenotype has been assessed with various self-reported questionnaires, which are prone to possible recall bias and a lack of diagnostic accuracy. The primary purpose of this study was to define the low physical activity domain of the frailty phenotype using accelerometer-based measurement and to evaluate the internal construct validity among older community-dwellers. Secondly, we examined potential correlates of frailty in this population.

**Methods:**

We conducted a cross-sectional study of 1,527 community-dwelling older men and women aged 65 and over. Data were drawn from the baseline survey of the Sasaguri Genkimon Study, a cohort study carried out in a west Japanese suburban community. Frailty phenotypes were defined by the following five components: unintentional weight loss, low grip strength, exhaustion, slow gait speed, and low physical activity. Of these criteria, physical activity was objectively measured with a tri-axial accelerometer. To confirm our measure’s internal validity, we performed a latent class analysis (LCA) to assess whether the five components could aggregate statistically into a syndrome. We examined the correlates of frailty using multiple stepwise logistic regression models.

**Results:**

The estimated prevalence of frailty was 9.3% (95% confidence intervals, CI, 8.4-11.2); 43.9% were pre-frail (95% CI, 41.5-46.4). The percentage of low physical activity was 19.5%. Objectively-assessed physical activity and other components aggregated statistically into a syndrome. Overall, increased age, poorer self-perceived health, depressive and anxiety symptoms, not consuming alcohol, no engagement in social activities, and cognitive impairment were associated with increased odds of frailty status, independent of co-morbidities.

**Conclusions:**

This study confirmed the internal construct validity of the frailty phenotype that defined the low energy expenditure domain with the objective measurement of physical activity. Accelerometry may potentially standardize the measurement of low physical activity and improve the diagnostic accuracy of the frailty phenotype criteria in primary care setting. The potential role of factors associated with frailty merits further studies to explore their clinical application.

**Electronic supplementary material:**

The online version of this article (doi:10.1186/s12877-015-0037-9) contains supplementary material, which is available to authorized users.

## Background

Frailty has been recognized as a biological syndrome [1]. It is theoretically defined as a clinically recognizable state of increased vulnerability to stressors, characterized by a decreased reserve capacity to maintain homeostasis resulting from an age-related cumulative decline across multiple physiologic systems [1,2]. Frailty confers an increased risk of adverse health outcomes, including falls, delirium, disability, hospitalization, long-term care, and mortality [3,4]. The incidence and prevalence of frailty are expected to increase with population aging, which consequently poses a great challenge to public healthcare and social care systems as demands for medical and care resources increase [5]. Therefore, early screening for frailty in routine clinical practice, especially in primary care settings, is of great significance considering its high prevalence, reversibility, and prognostic value [6,7].

The best evidence-based process to detect frailty and grade its severity is comprehensive geriatric assessment, but this is a resource-intensive process [1,7]. Although there is no universal consensus regarding specific operational criteria in different practice settings, two main operational definitions receiving broad acceptance are the frailty phenotype proposed and validated by Fried and colleagues in the Cardiovascular Health Study (CHS) [3] and the Frailty Index proposed and validated by Rockwood and colleagues in the Canadian Study of Health and Aging [8]. The Fried frailty phenotype is the most commonly used definition in community settings worldwide [9]. Compared to the Frailty Index, the frailty phenotype has been deemed more suitable for the immediate identification of non-disabled elders who are at increased risk for negative events, such as non-institutionalized community-dwellers [10,11].

The CHS frailty phenotype defines the presence of frailty and pre-frailty using five core components of the frailty cycle: unintentional weight loss, low grip strength, exhaustion, low gait speed, and low physical activity. In this measure, the presence of three or more components indicates frailty, one to two components designates pre-frailty, and zero components specify that the individual is not frail. Of these five components, low physical activity has been assessed in previous studies using questionnaires, which seemingly are feasible for routine practice, but prone to possible recall bias and a lack of diagnostic accuracy and comparability between different questionnaires. Specifically, for the frailty phenotype, the physical activity energy expenditure was assessed with the Minnesota Leisure Time Activity questionnaire, which does not capture physical activities in contexts other than specific leisure physical activities included in the questionnaire [3]. Furthermore, many studies have used various questionnaires containing different kinds of leisure physical activities from those of the CHS [12-15]. The measurement of the low physical activity domain has not been standardized, which to some extent hinders the widespread application of the frailty phenotype in primary care practice. Thus, we addressed this issue in our study. We defined the low physical activity domain of the frailty phenotype using accelerometer-based measurement to detect frailty and evaluated whether our measures could statistically aggregate into a syndrome on their own, among older community-dwellers in a suburban area in Japan. Secondly, we examined correlates of frailty across a constellation of social, psychological, environmental, and health-related factors.

## Methods

### Study population

Cross-sectional data were derived from the baseline survey of the Sasaguri Genkimon cohort study, an ongoing population-based prospective observational study [16]. The cohort was recruited from the town of Sasaguri, a suburb of the Fukuoka metropolitan area on Japan’s Kyushu Island. It is characterized as a region of low population mobility and a conventional Japanese lifestyle. The population of the town was 31,606 in January 2011 at the time of baseline survey. Based on data from the national census, the distributions of age, gender, education, and occupation in Sasaguri and for the whole of Japan are shown in supplemental Figure 1 (see Additional file 1).

**Figure 1:**
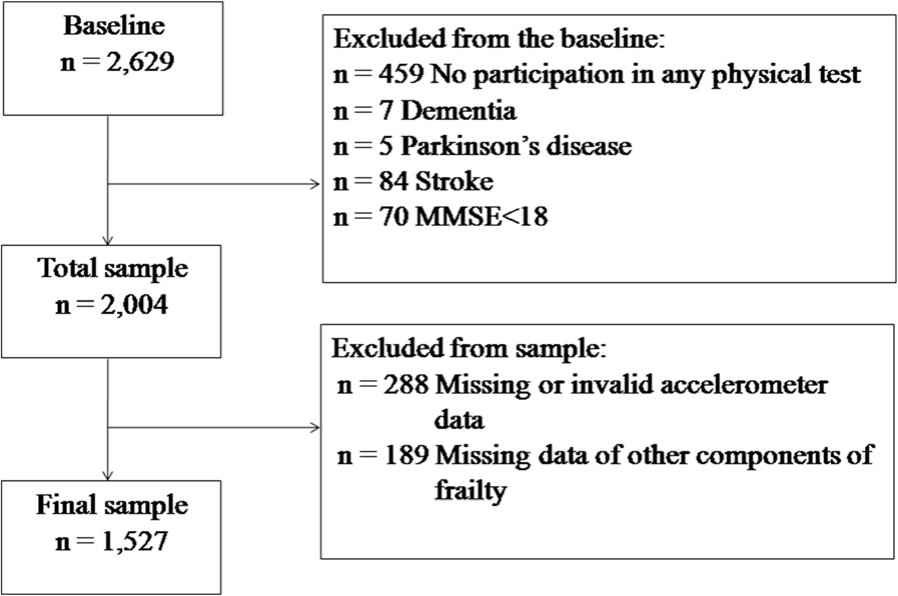
**Assembly of the study sample.**

The inclusion criterion was all primary residents aged 65 years and older and the exclusion criterion was inhabitants placed in residential long-term care, as identified by the national long-term care insurance system. There were 4,979 potential participants, representing 15.7% of the residents in this district. We contacted all potential participants by sending brochures and questionnaires by mail, except for those who had died or moved out of the district (n = 66) since the time of baseline measurements. Of the 4,913 individuals we contacted, 2,629 completed questionnaires, for a response rate of 53.5%. Individuals who did not respond were older (74.1 ± 7.1 vs. 73.5 ± 6.2, *p* = 0.002) but there was no gender difference for the respondents (*p* = 0.92). For the present study, we excluded those individuals who did not participate in any physical tests. In cases where frailty could potentially be a consequence of a single condition, we excluded subjects with a history of dementia, Parkinson’s disease, stroke, or a Mini-mental State Examination (MMSE) score <18. This exclusion was based on the CHS exclusion criteria [3]. Individuals with missing or invalid accelerometer data were also excluded. Among those who provided accelerometer data, 89.8% were adherent to the accelerometer protocol. The final sample consisted of 1,527 older men (n = 593) and women (n = 934) who had complete data for the other components of frailty (Figure 1). Comparisons of the characteristics between the excluded and included sample in this study were conducted (see Additional file 2).

### Measures

#### Operational definition of the frailty phenotype

All five of the original components of the CHS frailty phenotype, as well as their methodology to produce population specific cut-off points were retained in our study. The operational definition of each component was as follows (see details in Table 1). Individuals with three, and one to two affected components of the frailty measures were respectively considered as frail and pre-frail (intermediate frailty status) and those without any affected components were considered not frail. Of note, we measured low energy expenditure of physical activity objectively with a tri-axial accelerometer (Active Style Pro, HJA350-IT, Omron Healthcare, Co. Ltd, Kyoto, Japan) for at least one week. The accelerometer data are known to be more accurate than estimates from self-reported questionnaires and increasingly diffused in the general population [17]. Low physical activity was defined as scoring in the lowest 20% of energy expenditure of physical activity per day, stratified by gender. Data were quantified as kilocalories per kilogram of body weight expended per day (kcal/kg/day). A valid day was defined by wearing the tri-axial accelerometer for more than 600 minutes. Participants with ≥ 3 valid days were eligible for all analyses.

**Table 1:** **Operational definition of frailty phenotype in the Sasaguri Genkimon Study**

	**Definition**
Shrinking	Unintentional weight loss > 2–3 kg in the prior 6 months.
Weakness	Grip strength in the lowest 20%, stratified by gender and BMI (kg/m^2^)
Male	≤25.00 kg for BMI < 18.5, ≤ 30.00 kg for 18.5 ≤ BMI < 25, ≤ 31.50 kg for 25 ≤ BMI < 30, ≤ 33.00 kg for BMI ≥ 30
Female	≤17.50 kg for BMI < 18.5, ≤ 19.50 kg for 18.5 ≤ BMI < 25, ≤ 20.50 kg for BMI 25 ≤ BMI < 30, ≤ 19.75 kg for BMI ≥ 30
Exhaustion	Positive answer to either of two self-reported questions. Participants were asked how they felt in last one month: “Did you feel that everything you did was an effort?”, “Did you feel exhausted without any reason?”
Slowness	Time of 5-metre walk test at one’s maximum waking speed in the highest 20%, stratified by gender and standing height (gender-specific cutoff: a medium height).
Male	Time ≥ 3.56 s for height < 162.0 cm or Time ≥ 3.21 s for height ≥ 162.0 cm
Female	Time ≥ 4.25 s for height < 148.7 cm or Time ≥ 3.61 s for height ≥ 148.7 cm
Low physical activity	Lowest 20% of energy expenditure of physical activity by a triaxial accelerometer; quantified as kilocalories/kg (body weight), stratified by gender.
Male	≤6.20 kcal/kg/day
Female	≤7.13 kcal/kg/day
Overall frailty status	Non-frail: 0 affected component. Pre-frail: 1–2 affected components. Frail ≥ 3 affected components.

Shrinking was defined as unintentional weight loss > 2–3 kg in the previous 6 months. This threshold is commonly accepted in Japan and was originally used as an indicator of nutrition for identifying vulnerable community-dwelling older adults in the long-term care insurance system by the Japan Ministry of Health, Labour and Welfare [18,19]. This is similar to the original CHS definition of >10 lbs (4.5 kg) in the year prior [3]. Weakness was defined as scoring in the lowest 20% of grip strength, measured by a handheld dynamometer (GRIP-D, T.K.K. 5401; Takei Scientific Instruments Co. Ltd, Niigata, Japan), and was stratified by gender and body mass index (kg/m^2^). A measurement was taken for each hand, alternating between hands, and repeated again. We then averaged the greater values for both hands. Exhaustion was indicated by a positive answer to either of the following two self-reported questions. Participants were asked how they felt in the last one month: “Did you feel that everything you did was an effort?” and “Did you feel exhausted without any reason?” Slowness was identified as scoring in the slowest 20% of gait speed, based on the time for a 5-meter walking test at one’s maximum walking speed, stratified by gender and standing height. Gait speed was measured by a standard test procedure using a stop watch, as we have previously described elsewhere [20]. Participants were instructed to walk eleven meters at their maximum speed, starting from a motionless standing position. The time was recorded between the third meter and the eighth meter.

#### Socio-demographic and socio-psychological variables

A questionnaire captured socio-demographic information about educational attainment (years of formal education), income status (very poor, poor/fair, or good), living alone (yes/no), employment status (yes/no), and housing tenure (owned/mortgaged, rented, or other). Participants’ social networks were measured with the Japanese version of the Lubben Social Network Scale (LSNS-6), which is used worldwide as a screening tool for social isolation in community-dwelling elderly, and a cut-off score of 12 was adopted as recommended [21]. Self-perceived health was rated with a four-point scale asking how the respondent would rate one’s general health. The possible answers were as follows: poor, fair, good, and very good. Respondents were categorized into two groups: poor/fair and good/very good. The Japanese version of the Kessler Psychological Distress Scale (K6) was used to measure depressive and anxiety symptoms. The K6 has been increasingly used in community settings with an optimal cut-off score of > 4 indicating depressive and anxiety symptoms in Japan [22].

#### Health behavioral variables and comorbidities

With respect to health behaviors the questionnaire also inquired whether participants currently smoked or drank and if they did habitual exercise, or had any hobbies (e.g., music, painting, gardening, writing, reading, photography, pottery), as well as about their frequency of going outdoors (once a week or less/more than once a week). The question regarding engagement in any social activity (e.g., clubs for the elderly, volunteer work, religion-related activities, group activities, community activities, and business or professional activities) was answered with “yes/no.” Self-reported medical history data on having been diagnosed with chronic diseases (high blood pressure, chronic heart disease, hyperlipidemia, diabetes mellitus, minor trauma fracture, depression, chronic pulmonary disease, digestive disease, chronic renal disease, osteoarthritis or rheumatism, and cancer) were recorded.

#### Cognitive, physical, and social function capacity variables

Measures of function capacity included cognitive function, instrumental activities of daily living (IADLs), intellectual activity, and social role limitations. Cognitive function was measured using the MMSE with a cut-off point of 23/24 points indicating cognitive impairment [23]. IADLs, intellectual activity, and social role limitation were measured with the 13-item Tokyo Metropolitan Institute of Gerontology Index of Competence, which focuses on the competence to perform tasks in studies of elderly community residents [24]. Negative responses indicating inability to perform a specific task or needing assistance with it were identified as a disability in that task.

#### Ethical considerations

This cohort study protocol was approved by the Institutional Review Board of the Institute of Health Science Center, Kyushu University and conducted in accordance with the Declaration of Helsinki. Written informed consent was obtained from all participants.

#### Statistical analyses

To confirm our measure’s internal construct validity, we performed latent class analysis (LCA) to assess whether the five components could aggregate statistically into a model. The goal of LCA is to identify clusters of similar type of observations, estimating the characteristics of these latent groups [25]. The LCA hypothesis was that the population of older community-dwellers could be stratified into subpopulations characterized by sentinel patterns of aggregation of the frailty components with elimination of all confounding between the five frailty components [26]. We conducted the LCA in R version 3.1.2, using latent class analysis package version 1.4 (http://dlinzer.github.com/poLCA). We coded the five components as dichotomous variables to make a dataset for the LCA and computed the observed frequencies of 32 possible patterns of combinations of the frailty criteria. The LCA package in R used expectation-maximization and Newton–Raphson algorithms to find maximum likelihood estimates of model parameters. The appropriate number of latent classes was selected by comparing goodness-of-fit of models, including the most widely used parsimony measures of model fit: the Bayesian information criterion (BIC), Akaike information criterion (AIC), and Pearson’s χ^2^ [25]. Models that minimize values of the BIC and/or AIC were preferred.

Characteristics of participants were summarized by frailty status with means and standard deviation (SD) for continuous variables and frequencies with 95% confidence intervals (CIs) for categorical variables. Prevalence of frailty status was computed as percentages with 95% CIs. For associations of frailty with all independent variables, the p-value for the trend was assessed using the Cochran-Mantel-Haenszel test. Odds ratios and 95% CIs for each factor were calculated using univariate logistic analyses and mutually adjusted multivariate logistic analyses with backward-elimination. Multicollinearity between independent variables was ruled out by a variance inflation factor (VIF) test, with a value less than 2 indicated as appropriate. To exclude the possible confounding influence of co-morbidities on the associations between independent variables and frailty, we adjusted for co-morbidities that were significantly associated with frailty by bivariate analyses: minor trauma fracture, high blood pressure, chronic heart disease, diabetes, chronic pulmonary disease, digestive disease, and osteoarthritis or rheumatism. Finally, to elucidate the potential influence of gender on correlates of frailty, we stratified the multivariate model by gender. The multivariate model was evaluated with the Hosmer-Lemeshow goodness-of-fit test and Nagelkerke’s coefficient of determination. All statistical analyses were performed using SAS version 9.3 (SAS Institute Inc., Cary, N.C., USA). The statistical significance was set at *p* < 0.05.

## Results

### Distribution of sample characteristics and prevalence of frailty

In this study, participants were 65 to 93 years of age, with a mean age (SD) of 73.3 (6.0) years and 38.8% were men. The mean (SD) of educational attainment was 11.1 (2.5) years.

**Figure 2:**
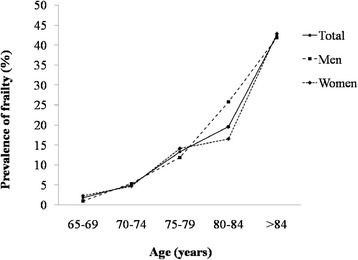
**Estimated prevalence of frailty by gender and age in the Sasaguri Genkimon Study.**

The percentages of frailty components were 19.5% for low physical activity, 14.8% for unintentional weight loss, 18.6% for low grip strength, 18.3% for exhaustion, and 17.1% for slow gait speed (Table 2). The mean (SD) length of accelerometer wearing time was 6.9 (1.6) days. In regards to the prevalence of frailty, 43.9% of the participants were identified as pre-frail (intermediate frailty status). The rates of frailty stratified by gender were 9.3% for both men and women. Figure 2 shows the prevalence of frailty by gender and age group. The prevalence of frailty increased considerably with each successive 5-year age grouping. The two curves representing men and women were similar, whilst the percentage of frailty increased dramatically starting in the 75–79 age group for men and the 80–84 age group for women.

**Table 2:** **Frailty phenotype components in percentages**

	**Total n = 1527**	**Men n = 593**	**Women n = 934**
Frequency of individual criterion,%			
Shrinking	14.8	16.5	13.7
Weakness	18.6	17.9	19.1
Exhaustion	18.3	16.9	19.3
Slowness	17.1	16.9	17.2
Low physical activity	19.5	19.1	19.8
Number of frailty criteria present,%			
0	46.8	47.1	46.8
1	29.9	29.9	29.9
2	14.1	13.8	14.2
3	7.0	7.4	6.8
4	2.2	1.9	2.4
5	0.1	0.0	0.2
Prevalence of frailty status,%			
Pre-frail	43.9	43.7	44.1
Frail	9.3	9.3	9.3

### Internal construct validity of frailty criteria

We found that the one-class latent model did not produce adequate expected frequencies of the observed frailty patterns. Both two-class and three-class latent models demonstrated a good model fit (Pearson Chi square *p* = 0.43 and 0.53), indicating that our measures of the frailty phenotype, which defined the low physical activity domain with accelerometer-based measurement, could be aggregated statistically into a syndrome and demonstrated satisfactory internal validity (Table 3). Comparisons of the maximum log-likelihood, Akaike Information Criterion (AIC), and Bayesian Information Criterion (BIC), did not reveal a better model fit for the three-class latent model than for the two-class latent model. In other words, our data did not suggest that the three-class phenotype was better than the two-class phenotype at stratifying people into subgroups characterized by sentinel patterns of aggregation of the frailty components. The increase of conditional probabilities of low physical activity from less-to-more frail classes within both the 2-class and 3-class models was similar to those of two other objectively-measured components: low grip strength and slow gait speed (data not shown).

**Table 3:** **Latent class analysis model fit of frailty criteria (n = 1,527)**

	**1-class model**	**2-class model**	**3-class model**
Maximum log-likelihood	−3553.3	−3432.6	−3427.9
AIC	7116	6887	6889
BIC	7143	6945	6980
Pearson χ^2^	262.5	21.5	13.0
	(*p* <0.001)	(*p* = 0.43)	(*p* = 0.53)

*Notes:* AIC = Akaike Information Criterion; BIC = Bayesian Information Criterion.

### Correlates of frailty

A trend test of all factors across frailty status revealed that those individuals who were identified as frail were older, less educated, more likely to be socially isolated, living alone, unemployed, currently not consuming alcohol, did not own or mortgage their own home, and had poorer self-perceived health and high levels of depressive and anxiety symptoms, than those who were not frail or pre-frail. They had significantly higher rates of functional limitations, including limitations in IADLs, intellectual activity, and social roles, as well as cognitive impairment. Those who did go outdoors less than once a week and did not engage in any hobbies, habitual exercise, or social activities were more likely to be frail and pre-frail (see Additional file 3).

The odds ratio with a one-year increment in age was 1.26 (95% CI, 1.22-1.31) for being frail and 1.09 (95% CI, 1.07-1.11) for being pre-frail, compared with the non-frail subgroups, meaning 26% higher odds of frailty and 9% higher odds of pre-frailty per unit increase of age (Table 4: Model 1). Participants who claimed to currently consume alcohol had lower odds of being frail. Living alone was associated with an increased odds ratio of pre-frailty, but it was not significant for frailty. Engagement in social activities showed a marked 53% reduction in the odds of frailty in the population. Those who reported poorer self-perceived health were almost four times more likely to be frail and two times more likely to be pre-frail. Higher depressive and anxiety symptoms were associated with significantly higher odds of frailty and pre-frailty. Cognitive impairment was associated with higher odds of being frail after adjusting for co-morbidities. An adjustment for comorbidities in the second model (Table 4: Model 2) did not alter the variables that showed independent statistically significant associations with frailty and pre-frailty. Finally, we analyzed correlates of frailty by stratifying the data by gender to examine its potential effect. Frailty was associated with living alone, not currently consuming alcohol, no engagement in social activities, social isolation, and cognitive impairment in women, but not in men (see Additional file 4). Adjustment for co-morbidities of each gender did not alter significant variables (data not shown).

**Table 4:** **Variables showing statistically significant associations with frailty status (n = 1459)**

	**Pre-frail**			**Frail**		
**Variables**	**Univariate OR (95% CI) for pre-frail vs. non-frail**	**Multivariate OR (95% CI) for pre-frail vs. non-frail**		**Univariate OR (95% CI) for frail vs. non-frail**	**Multivariate OR (95% CI) for frail vs. non-frail**	
		**Model 1**	**Model 2**		**Model 1**	**Model 2**
Age , 1 year increment	1.09 (1.07-1.11)*	1.09 (1.07-1.11)*	1.09 (1.07-1.11)*	1.25 (1.21-1.30)*	1.26 (1.22-1.31)*	1.26 (1.21-1.32)*
Living alone (reference: no)	1.56 (1.14-2.15)*	1.47 (1.03-2.10)*	1.57 (1.09-2.25)*	1.65 (0.99-2.73)	1.08 (0.58-2.03)	1.23 (0.63-2.38)
Current alcohol consumption (reference: no)	0.84 (0.68-1.04)	0.91 (0.70-1.20)	0.91 (0.70-1.18)	0.50 (0.34-0.75)*	0.56 (0.33-0.93)*	0.54 (0.32-0.92)*
Engagement in social activities (reference: no)	0.69 (0.54-0.90)*	0.78 (0.58-1.05)	0.78 (0.58-1.05)	0.39 (0.27-0.58)*	0.49 (0.30-0.81)*	0.47 (0.28-0.78)*
Self-perceived health (reference: good/very good)	2.75 (2.04-3.69)*	1.94 (1.40-2.69)*	2.00 (1.42-2.81)*	7.39 (4.92-11.11)*	4.79 (2.90-7.91)*	3.69 (2.17-6.28)*
Depressive and anxiety symptoms (K6), 1 unit increment	1.24 (1.19-1.28)*	1.23 (1.17-1.28)*	1.23 (1.18-1.29)*	1.41 (1.34-1.49)*	1.38 (1.29-1.46)*	1.39 (1.30-1.48)*
Cognitive impairment (reference: MMSE ≥ 24)	2.32 (1.29-4.18)*	1.84 (0.95-3.57)	1.81 (0.93-3.54)	6.33 (3.20-12.50)*	2.36 (0.96-5.83)	2.73 (1.09-6.83)*

Note. The sample size decreased from 1527 to 1459 due to missing value of independent variables. *Significant association. OR = Odds ratio, 95% CI = 95% confidence interval, K6 = Kessler Psychological Distress Scale, MMSE = Mini-Mental State Examination. Model 1: Mutually adjusted, adjusted R^2^ = 33.6%; Model 2: further adjusted for co-morbidities, adjusted R^2^ = 36.3%.

## Discussion

In this report, we defined the low physical activity domain of the frailty phenotype with accelerometer-based measurement and confirmed the statistical aggregation of the five components of the frailty phenotype into a syndrome using LCA models. The accelerometer-based measurement of the low physical activity domain could potentially be beneficial in improving the diagnostic accuracy of the frailty phenotype and increasing its feasibility in primary care practice. We observed that frailty affected approximately one out of ten elderly adults aged 65 and over in this community-dwelling population, in which the care burden of frailty is the focus of exponentially rising demands for public healthcare resources. We also found significant associations between frailty status and age, living alone, self-perceived health, depressive and anxiety symptoms, current alcohol consumption, engagement in social activities, and cognitive impairment, independent of co-morbidities.

The CHS frailty phenotype has been shown to have satisfactory internal validity in the Canadian Study of Health and Aging [10]. Prior LCAs of the frailty phenotype have been performed in the Women’s Health and Aging Study (WHAS) [26] and the Survey of Health, Ageing and Retirement in Europe [27] and have demonstrated satisfactory internal validity. Likewise, using LCA, we found that the five frailty criteria aggregated statistically into a syndrome. We further observed that the estimated probabilities of low physical activity exhibiting within latent frail classes were similar to those of two other objectively-measured components, regardless of whether subjects were stratified into two or three latent classes. These results suggest that our measures have satisfactory construct validity and could be used in this population. Additionally, our results did not conclude that classifying subjects into three subgroups (or classes) was better than two subgroups in characterizing the population. However, the CHS frailty definition of three phenotypes has been reported to have acceptable criterion validity as it identifies a profile of adverse health outcomes [3,10,13,26].

Our study established a frailty prevalence of 9.3%, which is in line with previous studies that defined frailty using Fried’s criteria [5]. Of note is that a large scale population-based survey in Japan reported a frailty prevalence of 11.3% based on the Fried phenotype among community-dwelling adults aged 65 years and older, which was slightly higher than the prevalence in our study [15]. Perhaps the discrepancy could be explained by the fact that they measured physical activities based on self-reported binary questions or that they did not use the lowest quintile approach for cut-off points. In the present study, we defined low physical activity with objective measurement and the percentages of low physical activity were 19.1% for men and 19.8% for women, which were generally lower than what was reported in the previous study. Many previous studies have reported proportions of low physical activity that range widely from 20% to 30% [12,13,26,28]. The large discrepancies between previous studies can at least be partly explained by the various methods used to measure physical activity, ranging from validated questionnaires to two simple questions. Limiting comparisons to studies that calculated energy expenditure with validated questionnaires and that used the lowest quintile approach seems likely to ensure that at least the populations identified are similar. However, essentially different questionnaires capture different types of leisure physical activities, for example, only six of the 18 leisure activities considered in the CHS were evaluated in the WHAS [26], suggesting that different populations may be characterized as having low physical activity, even if the proportion of low physical activity would be similar, with respect to people’s preferences for these activities.

The agreement between accelerometer- and questionnaire-based measurement of physical activity is relatively poor [29]. The cut-off points of energy expenditure of physical activity in this study were 6.20 kcal/kg/day for men and 7.13 kcal/kg/day for women. Obviously, the estimates of energy expenditure were much higher than those in the CHS, since tri-axial accelerometers are capable of recording energy expenditure derived from a variety of daily physical activities rather than specific physical activities. Although accelerometer-based measurement may be less comparable to existing or historical cohorts, this objective measure of low physical activity may potentially standardize measurement in future cohorts and improve diagnostic accuracy of the frailty phenotype. In addition, the objective measurement of physical activity can be administered by non-professionals, which could possibly raise the feasibility of the Fried frailty criteria in primary care settings. Commercial products of tri-axial accelerometer devices have been increasingly distributed and available in the general population after validation, even in the form of smartphone applications [30].

It is intriguing that we found that the prevalence of frailty was similar in both genders. This finding was consistent with some reports [14,31], while a systematic review showed a greater difference in the weighted prevalence of frailty between men and women (9.6% vs. 5.2%) [5]. The lack of a gender difference could be partly explained by the differences between North American or European and Japanese populations. Alternatively, there was a higher prevalence of frailty among men in our study than in other international studies. The age distribution for Japanese elderly men is different than for men from other nations: Japanese elderly men (+65 years) live longer and the proportion of the oldest old (+80 year) among elderly men is higher than those in many other nations (https://www.cia.gov/library/publications/resources/the-world-factbook/geos/ja.html). The age distribution for elderly men in our study was similar to the national age distribution, as shown in Additional file 1. Moreover, Japanese elderly men have reported a similar prevalence of sarcopenia with that of women [32], which plays a central role in the pathogenesis of frailty [3].

We found many similarities between the results in our studies and those reported in previous research [5], we found that the rate of frailty increased dependent on age after adjusting for co-morbidities. Frail individuals reported poorer self-perceived health [6]. A possible reason for is that as the level of frailty increases, so does the tendency to rate their health poorly [31,33]. Although we used a psychological distress scale to measure depressive and anxiety symptoms in our study, our result, which indicated a significant association with frailty, is in agreement with findings reported in previous studies using other depression measures [14,34,35]. Living alone, another factor that has been found to be related to frailty, is related to poorer nutrition, which is a cardinal component of frailty [3]. The diversity of social ties might exert a beneficial effect on frailty [34,36], while living alone may indicate poorer social ties [21].

In agreement with some previous studies [6], we observed that frailty was unrelated to socioeconomic status, such as education, income, employment status, or house tenure. This may be explained by the universal health coverage of social health insurance in Japan, especially for the oldest elders, and by the equity in social economic conditions adequate to health maintenance [37]. The observed associations between cognitive impairment and frailty could be explained by several mechanisms, such as Alzheimer’s disease pathology, hormone dysregulation, and impaired nutrition [38]. Concordant with previous studies [12,36], we also observed that current alcohol consumption was associated with lower odds of frailty. The association may be explained by an avoidance of alcohol [39] or a decrease of alcohol-related socialization among those who were frail, in line with Japanese culture that people commonly believe drinking alcohol facilitates socialization and mutual understanding between individuals [40]. Older adults who engaged in social activities were less likely to become frail. Frequent engagement in social activities could help to maintain physical and mental fitness [41] and then compensate for age-related decline in reserve and function. Another explanation is that withdrawal from social activities could be a behavioral precursor of frailty. These findings favored the notion that an overt state of frailty may be preceded by behavioral adaptation, such as withdrawal from social activities, made in response to declining physiologic reserve and capacity [2]. Early detection of frailty and pre-frailty before decreased reserves become more pronounced helps to shift towards more appropriate goal-directed and individualized care provision. The potential role of the correlates of frailty and pre-frailty in prevention and intervention merits further studies to explore their clinical application, since frailty is a reversible process.

### Strengths and limitations

To our knowledge, this is the first attempt to date using a tri-axial accelerometer to define energy expenditure of physical activity for the frailty phenotype. We examined a wide range of potential correlates of frailty covering social, psychological, environmental, and health-related factors. This study also has several important limitations. The sample was not nationally representative. The cross-sectional design prevents conclusions of directional relationships. There might have been selection bias due to the relatively low participation rate. However, given the similar prevalence findings in our study and previous studies, we may extrapolate that potential response or selection biases would not tend to lead to underestimation or over-estimation in the prevalence of frailty in this study.

## Conclusions

In summary, we defined the low physical activity domain of the frailty phenotype with accelerometer-based measurement for detecting frailty. We confirmed that five frailty components can statistically aggregate into a syndrome, providing evidence for the internal construct validity of our measures. Objective measurement may potentially standardize the low physical activity component and improve diagnostic accuracy of the frailty phenotype. Frailty is prevalent in this community-dwelling population. We also found significant associations between frailty status and age, living alone, self-perceived health, depressive and anxiety symptoms, current alcohol consumption, engagement in social activities, and cognitive impairment, independent of co-morbidities. The potential role of those factors associated with frailty in the prevention and intervention of frailty and pre-frailty merits further studies to explore their clinical application.

## Additional files

Additional file 1:
**Comparisons of age, gender, education and occupational distribution in the Sasaguri town and in the whole Japan in 2010 (national census).**
Additional file 2:
**Comparisons between the excluded and included sample in this study.**
Additional file 3:
**Distribution of characteristics of the sample by frailty status.**
Additional file 4:
**Variables showing independent statistically significant associations with frailty status by gender.**

## Abbreviations

CHS: Cardiovascular health study
MMSE: Mini-mental state examination
BMI: Body mass index
LSNS: Lubben social network scale
K6: Kessler psychological distress scale
IADL: Instrumental activities of daily living
LCA: Latent class analysis
AIC: Akaike information criterion
BIC: Bayesian information criterion
SD: Standard deviations
95% CI: 95% Confidence interval
OR: Odds ratio
WHAS: Women’s health and aging study
