# Supplementary material for: Screening for frailty phenotype with objectively-measured physical activity in a west Japanese suburban community: evidence from the Sasaguri Genkimon Study
Source: BMC Geriatr. 2015 Apr 2;15:36. doi: 10.1186/s12877-015-0037-9 (PMC4391124; doi:10.1186/s12877-015-0037-9)
Supplement: Additional file 1: — Comparisons of age, gender, education and occupational distribution in the Sasaguri town and in the whole Japan in 2010 (national census). [file 12877_2015_37_MOESM1_ESM.pdf]

[A] Age distributions by gender (Census 2010)

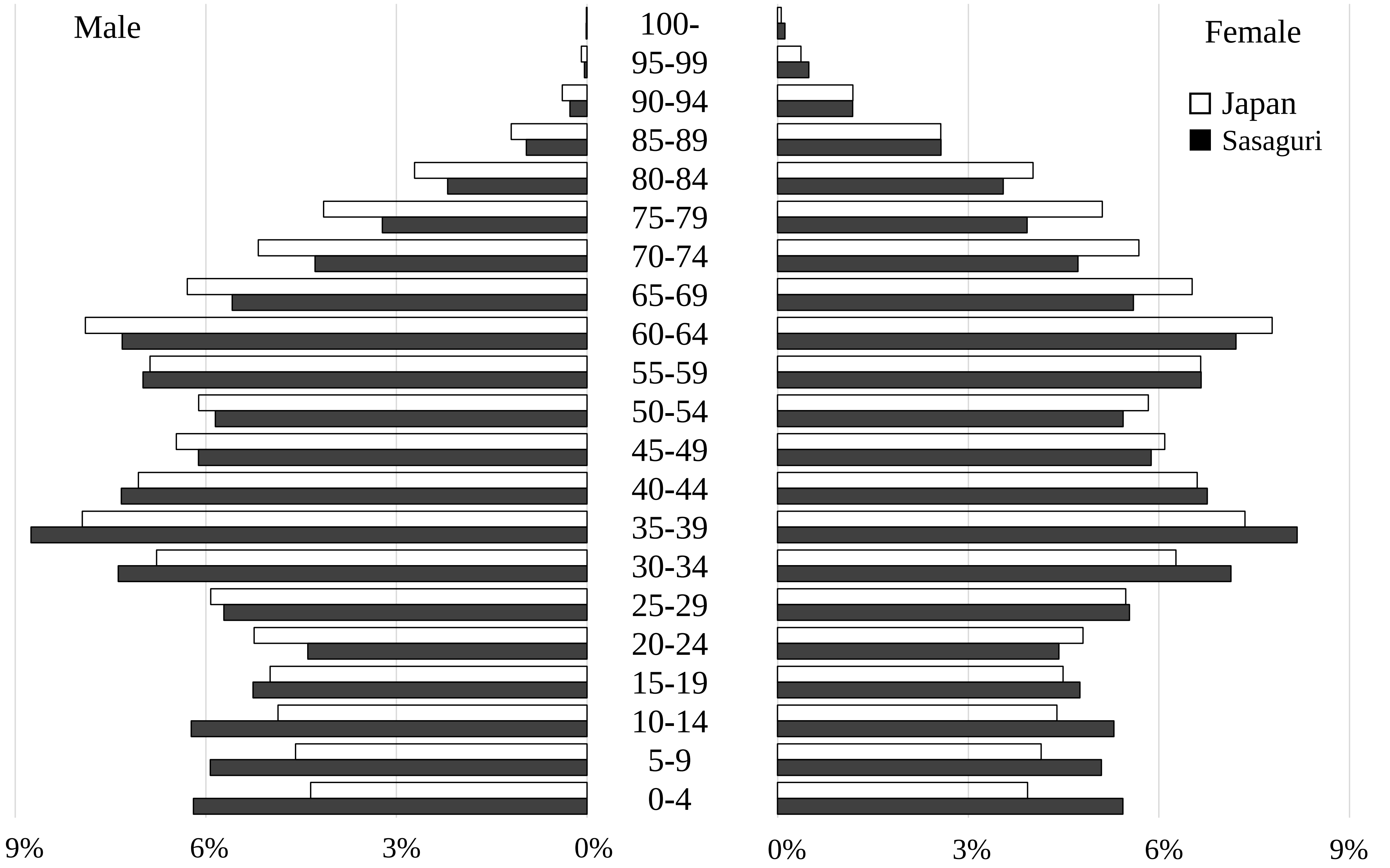

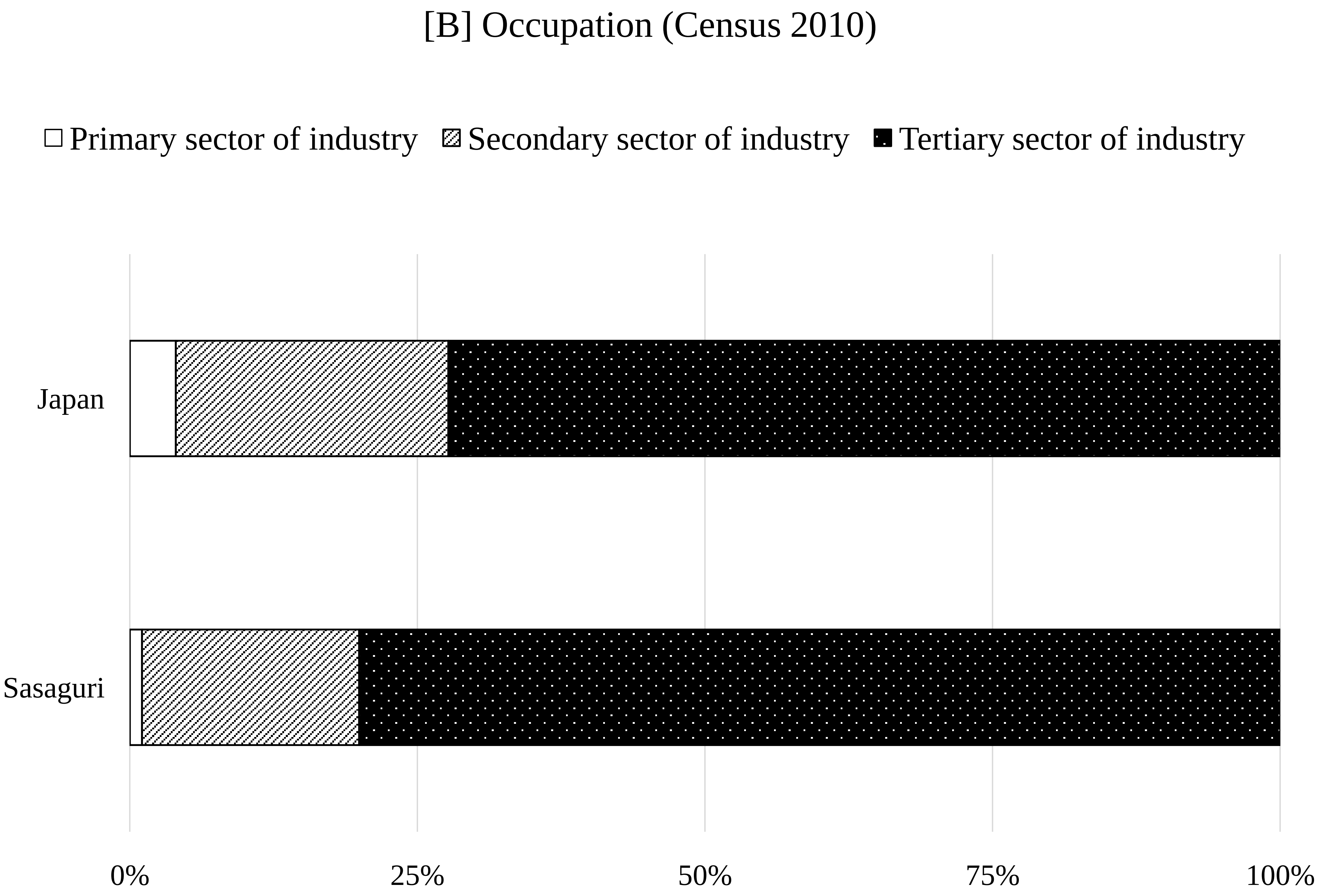
[B] Occupation (Census 2010)
Primary sector of industry
Secondary sector of industry
Tertiary sector of industry
Japan
Sasaguri
0%
25%
50%
75%
100%

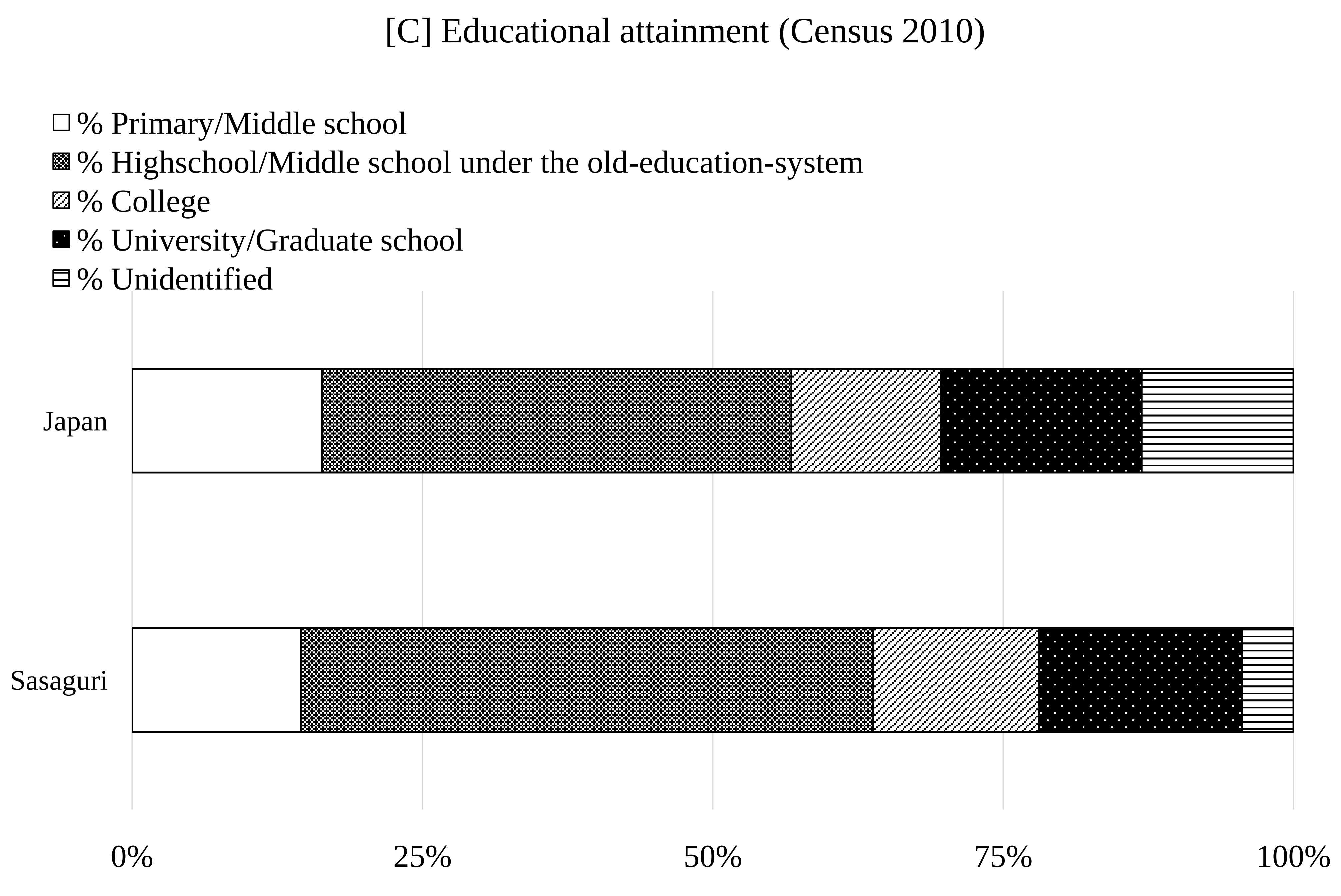
[C] Educational attainment (Census 2010)
% Primary/Middle school
% Highschool/Middle school under the old-education-system
% College
% University/Graduate school
% Unidentified
Japan
Sasaguri
0%
25%
50%
75%
100%
