# Supplementary material for: Screening for frailty phenotype with objectively-measured physical activity in a west Japanese suburban community: evidence from the Sasaguri Genkimon Study
Source: BMC Geriatr. 2015 Apr 2;15:36. doi: 10.1186/s12877-015-0037-9 (PMC4391124; doi:10.1186/s12877-015-0037-9)
Supplement: Additional file 2: — Comparisons between the excluded and included sample in this study. [file 12877_2015_37_MOESM2_ESM.docx]

Additional file 2: Comparisons between the excluded and included sample in this study

|  | Excluded | Included | *p* value |
| --- | --- | --- | --- |
|  | n=1,102 | n=1,527 |  |
| Gender (% men) | 50.3 | 38.8 | <.001 |
| Living alone, % | 10.8 | 13.2 | 0.06 |
| Income status (% very poor/poor) | 65.0 | 60.2 | 0.02 |
| Housing tenure (% owned/mortgaged) | 13.8 | 9.9 | 0.002 |
| Self-perceived health (% poor/fair) | 24.1 | 20.1 | 0.02 |
| Socially isolated (LSNS<12) | 22.9 | 18.1 | 0.01 |
| Currently employed, % | 24.3 | 17.2 | <.001 |
| Current smoking, % | 12.7 | 7.8 | <.001 |
| Current alcohol consumption , % | 44.2 | 38.9 | 0.01 |
| Going outdoors (% rarely) | 9.7 | 4.3 | <.001 |
| Habitual exercise (% yes) | 55.2 | 61.4 | <.001 |
| Engagement in social activities (% yes) | 62.5 | 76.6 | <.001 |
| Having hobbies (% yes) | 78.6 | 84.7 | <.001 |
| IADLs limitations (% difficulty ≥ 1 tasks) | 15.0 | 8.3 | <.001 |
| Intellectual activity limitations (% difficulty ≥ 1 tasks) | 35.2 | 27.0 | <.001 |
| Social role limitations (% difficulty ≥ 1 tasks) | 50.4 | 39.4 | <.001 |
| Cognitive impairment (% MMSE score < 24) | 15.0 | 4.7 | <.001 |
|  | Mean (SD) | Mean (SD) |  |
| Age, years | 73.8 (6.5) | 73.3 (6.0) | 0.06 |
| Education, years | 11.0 (2.6) | 11.1 (2.5) | 0.31 |
| K6 score, points | 3.5 (3.8) | 3.2 (3.4) | 0.13 |
| Note. LSNS = Lubben Social Network Scale; IADLs= Instrumental Activities of Daily Living; K6 = Kessler Psychological Distress Scale; MMSE = Mini-Mental State Examination | | | |
