# Supplementary material for: Screening for frailty phenotype with objectively-measured physical activity in a west Japanese suburban community: evidence from the Sasaguri Genkimon Study
Source: BMC Geriatr. 2015 Apr 2;15:36. doi: 10.1186/s12877-015-0037-9 (PMC4391124; doi:10.1186/s12877-015-0037-9)
Supplement: Additional file 3: — Distribution of characteristics of the sample by frailty status. [file 12877_2015_37_MOESM3_ESM.docx]

Additional file 3: Distribution of characteristics of the sample by frailty status

|  | Non-frail | | | | Pre-frail | | | Frail | | |  |
| --- | --- | --- | --- | --- | --- | --- | --- | --- | --- | --- | --- |
| Characteristics | n | % | 95% CI | | n | % | 95% CI | n | % | 95% CI | *p* for trend |
| All | 714 | 46.9 | 44.3-49.3 | | 671 | 43.9 | 41.5-46.4 | 142 | 9.3 | 7.9-10.9 |  |
| **Socio-demographic, socio-psychological factors** | | | | | |  |  |  |  |  |  |
| Gender |  |  |  | |  |  |  |  |  |  | 0.88 |
| Men | 279 | 47.1 | 43.1-51.1 | | 259 | 43.7 | 39.7-47.7 | 55 | 9.3 | 7.2-11.9 |  |
| Women | 435 | 46.6 | 43.4-49.8 | | 412 | 44.1 | 41.0-47.3 | 87 | 9.3 | 7.61-11.4 |  |
| Age (years) |  |  |  | |  |  |  |  |  |  | <0.001 |
| 65-69 | 316 | 61.8 | 57.6-66.0 | | 186 | 36.4 | 32.3-40.7 | 9 | 1.8 | 0.9-3.3 |  |
| 70-74 | 220 | 52.0 | 47.3-56.7 | | 182 | 43.0 | 38.1-47.8 | 21 | 5.0 | 3.3-7.5 |  |
| 75-79 | 120 | 36.3 | 31.3-41.6 | | 167 | 50.5 | 45.1-55.8 | 44 | 13.3 | 10.1-17.4 |  |
| 80-84 | 47 | 24.8 | 19.3-31.5 | | 105 | 55.6 | 48.4-62.5 | 37 | 19.6 | 14.6-25.8 |  |
| ≥ 85 | 11 | 15.1 | 8.6-25.0 | | 31 | 42.5 | 31.8-53.9 | 31 | 42.5 | 31.8-53.9 |  |
| Living alone |  |  |  | |  |  |  |  |  |  | 0.01 |
| Yes | 75 | 37.1 | 30.8-44.0 | | 104 | 51.5 | 44.6-58.3 | 23 | 11.4 | 7.7-16.5 |  |
| No | 639 | 48.2 | 45.5-50.9 | | 567 | 42.8 | 40.2-45.5 | 134 | 8.9 | 7.6-10.6 |  |
| Education (years) |  |  |  | |  |  |  |  |  |  | <0.001 |
| < 11 | 432 | 54.6 | 51.1-58.1 | | 318 | 40.2 | 36.8-43.7 | 41 | 5.2 | 3.8-7.0 |  |
| ≥ 12 | 282 | 38.5 | 35.0-42.1 | | 350 | 47.8 | 44.2-51.4 | 101 | 13.8 | 11.5-16.5 |  |
| Income status |  |  |  | |  |  |  |  |  |  | 0.60 |
| Very poor/poor | 415 | 46.0 | 42.8-49.3 | | 404 | 44.8 | 41.6-48.1 | 56 | 9.2 | 7.5-11.3 |  |
| Fair/good | 286 | 48.0 | 44.0-52.0 | | 254 | 42.6 | 38.7-46.6 | 83 | 9.4 | 7.3-12.0 |  |
| Housing tenure |  |  |  | |  |  |  |  |  |  | 0.01 |
| Owned/mortgaged | 657 | 47.9 | 45.2-50.5 | | 595 | 43.3 | 40.7-46.0 | 121 | 8.8 | 7.4-10.4 |  |
| Rented/other | 56 | 37.3 | 30.0-45.3 | | 75 | 50.0 | 42.1-57.9 | 19 | 12.7 | 8.3-18.9 |  |
| Self-perceived health |  |  |  | |  |  |  |  |  |  | <0.001 |
| Poor/fair | 76 | 24.8 | 20.3-29.9 | | 165 | 53.8 | 48.2-59.2 | 66 | 21.5 | 17.3-26.4 |  |
| Good/very good | 638 | 52.4 | 49.6-55.2 | | 504 | 41.4 | 38.7-44.2 | 75 | 6.2 | 4.9-7.7 |  |
| Psychological distress | | |  | |  |  |  |  |  |  | <0.001 |
| Yes (K6 > 4) | 113 | 25.2 | 21.4-29.4 | | 254 | 56.6 | 52.0-61.1 | 82 | 18.3 | 15.0-22.1 |  |
| No | 601 | 55.8 | 52.8-58.7 | | 417 | 38.7 | 35.8-41.6 | 60 | 5.6 | 4.4-7.1 |  |
| Socially isolated |  |  | |  |  |  |  |  |  |  | <0.001 |
| Yes (LSNS < 12) | 85 | 30.9 | | 25.7-36.6 | 142 | 51.6 | 45.8-57.5 | 48 | 17.5 | 13.4-22.4 |  |
| No | 626 | 50.4 | | 47.6-53.2 | 525 | 42.3 | 39.6-45.0 | 91 | 7.3 | 6.01-8.9 |  |
| Currently employed |  |  | |  |  |  |  |  |  |  | <0.001 |
| Yes | 146 | 55.7 | | 49.7-61.6 | 107 | 40.8 | 35.1-46.9 | 9 | 3.4 | 1.8-6.4 |  |
| No | 566 | 45.0 | | 42.2-47.7 | 561 | 44.6 | 41.8-47.3 | 132 | 10.5 | 8.9-12.3 |  |
| **Health behaviors factors** | | | |  |  |  |  |  |  |  |  |
| Current smoking |  |  | |  |  |  |  |  |  |  | 0.57 |
| Yes | 56 | 47.5 | | 38.7-56.4 | 54 | 45.8 | 37.1-54.7 | 8 | 6.8 | 3.5-12.8 |  |
| No | 655 | 46.7 | | 44.1-49.3 | 613 | 43.7 | 41.2-46.3 | 134 | 9.6 | 8.1-11.2 |  |
| Current alcohol consumption |  |  | |  |  |  |  |  |  |  | 0.001 |
| Yes | 301 | 50.8 | | 46.7-54.8 | 254 | 42.8 | 38.9-46.9 | 38 | 6.41 | 4.7-8.7 |  |
| No | 413 | 44.3 | | 41.1-47.5 | 416 | 44.6 | 41.4-47.8 | 104 | 11.2 | 9.3-13.3 |  |
| Going outdoors |  |  | |  |  |  |  |  |  |  | <0.001 |
| frequently | 697 | 47.7 | | 45.2-50.3 | 637 | 43.6 | 41.1-46.2 | 126 | 8.6 | 7.3-10.2 |  |
| rarely | 16 | 24.6 | | 15.8-36.3 | 33 | 50.8 | 38.9-62.5 | 16 | 24.6 | 15.8-36.3 |  |
| Habitual exercise |  |  | |  |  |  |  |  |  |  | 0.01 |
| Yes | 468 | 49.9 | | 46.7-53.1 | 386 | 41.2 | 38.0-44.3 | 84 | 9.0 | 7.3-11.0 |  |
| No | 246 | 41.8 | | 37.9-45.8 | 285 | 48.4 | 44.4-52.4 | 58 | 9.9 | 7.7-12.5 |  |
| Engagement in social activities | | | |  |  |  |  |  |  |  | <0.001 |
| Yes | 579 | 49.5 | | 46.6-52.4 | 502 | 42.9 | 40.1-45.8 | 89 | 7.6 | 6.2-9.3 |  |
| No | 135 | 37.8 | | 32.9-43.0 | 169 | 47.3 | 42.2-52.5 | 53 | 14.9 | 11.5-18.9 |  |
| Having hobbies |  |  | |  |  |  |  |  |  |  | <0.001 |
| Yes | 635 | 49.1 | | 46.4-51.8 | 557 | 43.1 | 40.4-45.8 | 101 | 7.8 | 6.5-9.4 |  |
| No | 79 | 33.9 | | 28.1-40.2 | 113 | 48.5 | 42.2-54.9 | 41 | 17.6 | 14.2-23.0 |  |
| **Function factors** |  |  | |  |  |  |  |  |  |  |  |
| IADLs limitations (difficulty ≥ 1 tasks) | | | | |  |  |  |  |  |  | <0.001 |
| Yes | 41 | 32.5 | | 25.0-41.1 | 64 | 50.8 | 42.2-59.4 | 21 | 16.7 | 11.2-24.1 |  |
| No | 672 | 48.0 | | 45.4-50.6 | 607 | 43.4 | 40.8-46.0 | 121 | 8.64 | 7.3-10.2 |  |
| Intellectual activity limitations (difficulty ≥ 1 tasks) | | | | | |  |  |  |  |  | <0.001 |
| Yes | 166 | 40.3 | | 35.7-45.1 | 196 | 47.6 | 42.8-52.4 | 50 | 12.1 | 9.3-15.6 |  |
| No | 548 | 49.2 | | 46.3-52.1 | 474 | 42.6 | 39.7-45.5 | 92 | 8.3 | 6.8-10.0 |  |
| Social role limitations (difficulty ≥ 1 tasks) | | | | |  |  |  |  |  |  | <0.001 |
| Yes | 224 | 37.3 | | 33.6-41.3 | 295 | 49.2 | 45.2-53.2 | 81 | 13.5 | 11.0-16.5 |  |
| No | 488 | 52.8 | | 49.6-56.0 | 375 | 40.6 | 37.5-43.8 | 61 | 6.6 | 5.2-8.4 |  |
| Cognitive impairment (MMSE < 24) | | | | |  |  |  |  |  |  | <0.001 |
| Yes | 17 | 23.6 | | 15.3-34.6 | 36 | 50.0 | 38.8-61.3 | 19 | 26.4 | 17.6-37.6 |  |
| No | 697 | 47.9 | | 45.4-50.5 | 635 | 43.6 | 41.1-46.2 | 123 | 8.4 | 7.1-10.0 |  |
| Note. 95% CI= 95% Confidence Interval; LSNS = Lubben Social Network Scale; IADLs= Instrumental Activities of Daily Living; K6 = Kessler Psychological Distress Scale; MMSE = Mini-Mental State Examination | | | | | | | | | | | |
