# Supplementary material for: Screening for frailty phenotype with objectively-measured physical activity in a west Japanese suburban community: evidence from the Sasaguri Genkimon Study
Source: BMC Geriatr. 2015 Apr 2;15:36. doi: 10.1186/s12877-015-0037-9 (PMC4391124; doi:10.1186/s12877-015-0037-9)
Supplement: Additional file 4: — Variables showing independent statistically significant associations with frailty status by gender. [file 12877_2015_37_MOESM4_ESM.doc]

Additional file 4: Variables showing statistically significant and independent associations with frailty status by gender

|  | Pre-frailty | Frailty |
| --- | --- | --- |
| Variables | Multivariate OR (95% CI) for pre-frailty vs. non-frailty | Multivariate OR (95% CI) for frailty vs. non-frailty |
| **Female (n=934)** |  |  |
| Age, 1 year increment | 1.10 (1.07-1.13)* | 1.26 (1.20-1.33)* |
| Living alone (reference: no) | 1.64 (1.09-2.46)* | 1.11 (0.52-2.34) |
| Current alcohol consumption (reference: no) | 1.08 (0.76-1.54) | 0.34 (0.15-0.81)* |
| Engagement in social activities (reference: no) | 0.74 (0.50-1.09) | 0.41 (0.22-0.77)* |
| Socially isolated (reference: LSNS ≥ 12) | 1.47 (0.93-2.32) | 2.37 (1.20-4.70)* |
| Self-perceived health (reference: good/very good) | 1.93 (1.26-2.95)* | 4.98 (2.60-9.57)* |
| Psychological distress (K6), 1 unit increment | 1.22 (1.16-1.29)* | 1.37 (1.26-1.48)* |
| Cognitive impairment (reference: MMSE ≥ 24) | 3.61 (1.28-10.20)* | 4.76 (1.32-17.18)* |
| **Male (n=593)** |  |  |
| Age, 1 year increment | 1.07 (1.03-1.11)* | 1.27 (1.20-1.35)* |
| Self-perceived health (reference: good/very good) | 2.15 (1.28-3.62)* | 3.74 (1.64-8.54)* |
| Psychological distress (K6), 1 unit increment | 1.24 (1.15-1.34)* | 1.44 (1.29-1.60)* |

Note. *Significant association. OR = Odds ratio; CI = confidence interval; LSNS = Lubben Social Network Scale; K6 = Kessler Psychological Distress Scale; MMSE = Mini-Mental State Examination
